# An optimal search filter for retrieving systematic reviews and meta-analyses

**DOI:** 10.1186/1471-2288-12-51

**Published:** 2012-04-18

**Authors:** Edwin Lee, Maureen Dobbins, Kara DeCorby, Lyndsey McRae, Daiva Tirilis, Heather Husson

**Affiliations:** 1 Faculty of Health Sciences, University of Western Ontario, Arthur and Sonia Labatt Health Sciences Building, London, ON, HSB 200 N6A 5B9, Canada; 2 Faculty of Health Sciences, McMaster University, 1200 Main St. W, Hamilton, ON, Canada

## Abstract

**Background:**

Health-evidence.ca is an online registry of systematic reviews evaluating the effectiveness of public health interventions. Extensive searching of bibliographic databases is required to keep the registry up to date. However, search filters have been developed to assist in searching the extensive amount of published literature indexed. Search filters can be designed to find literature related to a certain subject (i.e. content-specific filter) or particular study designs (i.e. methodological filter). The objective of this paper is to describe the development and validation of the health-evidence.ca Systematic Review search filter and to compare its performance to other available systematic review filters.

**Methods:**

This analysis of search filters was conducted in MEDLINE, EMBASE, and CINAHL. The performance of thirty-one search filters in total was assessed. A validation data set of 219 articles indexed between January 2004 and December 2005 was used to evaluate performance on sensitivity, specificity, precision and the number needed to read for each filter.

**Results:**

Nineteen of 31 search filters were effective in retrieving a high level of relevant articles (sensitivity scores greater than 85%). The majority achieved a high degree of sensitivity at the expense of precision and yielded large result sets. The main advantage of the health-evidence.ca Systematic Review search filter in comparison to the other filters was that it maintained the same level of sensitivity while reducing the number of articles that needed to be screened.

**Conclusions:**

The health-evidence.ca Systematic Review search filter is a useful tool for identifying published systematic reviews, with further screening to identify those evaluating the effectiveness of public health interventions. The filter that narrows the focus saves considerable time and resources during updates of this online resource, without sacrificing sensitivity.

## Background

Systematic reviews have been integral to the evidence-informed practice movement [1-5] in the field of public health [6-9]. A systematic review consists of an examination of all of the primary studies on a topic, which includes searching for, collating, and assessing the studies, to establish conclusive evidence about a topic [10]. Systematic reviews present a more consistent and conservative estimate of the effect of interventions across a body of literature and as such, can have an important impact on program planning decisions in public health. However, public health decision makers state that finding and accessing systematic reviews related to public health continues to be a barrier to evidence-informed public health practice [11-16]. The field of public health can be defined as a combination of sciences, skills, and values that function through collective societal, legislative, and political activities. It involves both public and private programs, services, and institutions aimed at protecting and improving the health of all people, including preventing disease, promoting health and wellbeing, and prolonging life. When necessary, public health also engages in restoring the health of individuals, specified groups, populations or communities through mobilizing and engaging local, state, national, and international resources to assure the conditions in which people can be healthy [17-19]. In short, the field of public health is broad, and decision makers wear many hats, requiring evidence on a wide range of topics.

Public health practitioners have expressed a need for a single place where they can access reviews evaluating the effectiveness of interventions, have confidence in the methodological quality of the evidence, and access plain language review summaries with corresponding implications for policy and practice [20]. Health-evidence.ca is a free, searchable online registry of systematic reviews and meta-analyses evaluating the effectiveness of public health and health promotion interventions. This registry represents one component of a larger knowledge translation and exchange (KTE) [21] strategy that supports users in accessing and interpreting research evidence. KTE is a two-way process involving dialogue, interaction, and the sharing of knowledge and evidence between and among the producers and users of knowledge and research evidence. It is a broad term that is often used to include knowledge transfer, exchange, translation, dissemination, and diffusion. The target audience for health-evidence.ca is decision makers working in public health and health promotion at all levels (front line practitioners to senior management and policy makers in government). Public health decision makers need to find, assess and interpret research evidence quickly and easily if it is to inform program and policy decisions. Health-evidence.ca provides decision makers with easy access to public health-relevant, quality-appraised systematic reviews evaluating the effectiveness of public health interventions. The site is freely accessible and can be searched by selecting common public health indexing terms. Search results include links to published review abstracts and a rating of the methodological quality of each review. In addition, health-evidence.ca team members write evidence summaries for reviews of good methodological quality to summarize key findings and provide recommendations for policy and practice. A more complete description of this online resource has been published and is accessible at http://www.biomedcentral.com/1471-2458/10/496.

Health-evidence.ca was updated quarterly until 2012 and is now updated on a monthly basis. Updates consist of conducting monthly searches of relevant electronic databases, importing results into a bibliographic database management program, screening titles to identify relevant articles, retrieving potentially relevant articles and screening full document versions for inclusion. Included reviews must meet relevance criteria and must be systematic reviews that focus on public health, provide outcome data on the effectiveness of interventions, and include a documented search strategy.

As of February 2012, over 1,017,500 titles had been screened, yielding 2,450 relevant reviews. The large number of titles screened to reach the final, relevant set reflects the challenges of searching bibliographic databases for public health and health promotion literature. These challenges stem from the lack of a single database dedicated exclusively to public health and health promotion literature, requiring searches in multiple health (MEDLINE, EMBASE, CINAHL), science, and social science databases (BIOSIS, PsycINFO, SPORTDiscus, Sociological Abstracts). There are also several limitations inherent in searching these databases. For example, 33-44% of the journals identified by experts in the field as public health journals are not indexed in MEDLINE. These challenges are not limited to public health as others have encountered similar difficulties in searching for mental health content [23] and health services research literature [24]. A further challenge is identifying what is relevant to public health and health promotion practitioners, given that it is a dynamic field characterized by a wide scope of practice, defined regionally and changing constantly.

Along with the challenges of searching for public health and health promotion content, review literature, though rapidly growing, remains limited in volume when compared to primary studies. For example, over 700,000 articles were indexed in MEDLINE in 2010, of which approximately 2500 (0.36%) were health-related systematic reviews [25]. Currently, there is no single MEDLINE subject heading term for ‘systematic review’; this lack of an indexing term requires the end user to employ a Clinical Query developed to locate systematic reviews, or to screen very large sets of irrelevant articles in order to retrieve systematic reviews. MEDLINE does have an indexing term for ‘review’ however its application is very broad. Of the 19,430,768 articles currently indexed in MEDLINE as of February 13, 2012, 8.5% (1,656,583) [26] were indexed as reviews. Upon screening a small portion of this results set, it was evident that the majority were not systematic reviews, but rather literature reviews and overviews. While the MEDLINE indexing term ‘meta-analysis’ is useful for identifying systematic reviews, it only captures systematic reviews that use statistical software to combine the results of the included primary studies in a single pooled estimate of effect. However, meta-analyses represent a small portion of all reviews evaluating the effectiveness of public health interventions. For example, fewer than half of public health intervention reviews indexed on health-evidence.ca are meta-analyses, thus reliance on this text word to identify reviews is not sufficient. A combination of indexing terms is required to detect relevant reviews that can be captured in online databases such as MEDLINE. Thus, although it has been time-consuming, screening a high number of irrelevant articles has been necessary. Search filters, also referred to as “search hedges”, are “collections of search terms intended to capture frequently sought research methods such as randomized controlled trials, or other aspects of health care” [27]. While search filters for the retrieval of systematic reviews were being used by others for searching MEDLINE [19-31], EMBASE [32], and CINAHL [33], none had been used and tested for locating public health and health promotion reviews that we were aware of at the time of this project. These filters, including those targeting content-specific literature relevant to the subject of interest [24,25], provided guidance as we developed a systematic review filter for health-evidence.ca.

Prior to 2008, we used a Public Health (PH) search filter that was developed in collaboration with health science librarians at McMaster University. The Head of Public Services worked with one of the authors (KD) to systematically run and informally evaluate the results of various search strategies for retrieving systematic reviews and meta-analyses evaluating the effectiveness of public interventions in MEDLINE, EMBASE, CINAHL, PsycINFO, and Sociological Abstracts. Search strategies were assessed and improvements made based on findings. The resulting PH search filter consisted of two distinct components: 1) indexing terms and keywords referring to systematic review methods, combined with the Boolean ‘OR’ operator (systematic, meta analysis, review); and 2) indexing terms and keywords referring to public health content areas, combined with the Boolean ‘OR’ operator (community health services, education, health education, health promotion, prevention, preventive). The content and methods components were then combined using the Boolean ‘AND’ operator. Seventeen topic areas were included in the content component: addiction, adult health, chronic diseases, communicable disease and infection, community health, dental health, environmental health, food safety and inspection, injury prevention and safety, mental health, nutrition, parenting, physical activity, pregnancy, sexual education, sexually transmitted infections, and women’s health. This search strategy also made it more likely that we would capture articles for which established indexing terms did not exist such as social determinants of health and healthy communities.

Our PH search filter typically yielded a very high volume of results with very low precision. For example, between January 2006 and December 2007, of the 136,427 titles screened, 409 were relevant for the health-evidence.ca registry, or in other words, precision was 0.3%. In addition to using the PH search filter, more than 40 public health-relevant journals were hand searched annually, as well as the reference lists of all relevant reviews. Given this systematic search of the published review literature, we were reasonably confident that our retrieval methods were capturing a near complete set of relevant articles. We considered this set (the electronic database searches plus additional search strategies), the ‘gold standard’ for health-evidence.ca. A gold standard is “a set of relevant records against which a new search filter is tested and validated to determine how effective it is at retrieving particular types of records” [34]. While it is impossible to prove that the gold standard for health-evidence.ca identified all public health relevant systematic reviews, we are confident that this approach captured the vast majority of relevant reviews.

Given that the precision of the PH search filter was so low, we began to create an effective search filter that would decrease the total number of results retrieved, while maximizing the number of relevant results. The health-evidence.ca Systematic Review (SR) search filter we developed in 2008 was adapted from a previously-validated filter [30], which included the terms: MEDLINE.tw, systematic review.tw, meta-analysis.pt, combined with the Boolean OR operator. While this filter was highly specific, it captured less than 82% of articles identified by our gold standard set. To customize this filter to retrieve only those systematic reviews of interventions, the term ‘intervention’ was added as an indexing term. This is referred to as the development data set.

The MEDLINE version of our health-evidence.ca SR search filter included the following indexing terms, combined with the Boolean ‘OR’ operator: MEDLINE.tw, systematic review.tw, meta-analysis.pt, intervention$.ti. We slightly modified the filter for use in EMBASE and CINAHL due to differences in indexing terms between the various databases. The indexing terms systematic review.tw and intervention$.ti are viable in both EMBASE and CINAHL, therefore these terms were consistent across all three databases. However, in both EMBASE and CINAHL, meta-analysis was not an indexed publication type, and therefore the term meta-analysis was included as a keyword in the search filter for these two databases. Each database employs a unique controlled vocabulary, thus the search strategy is tailored to the database. For example, MEDLINE does not have a preferred search term for systematic review so that concept must be searched as a text word. EMBASE and CINAHL, however, do have a specific indexing term for systematic review, so that term is used when tailoring the search to those databases.

The objective of this paper is to report the results of our efforts to evaluate and validate the health-evidence.ca SR search filter for retrieving systematic reviews and meta-analyses that evaluate the effectiveness of interventions. First, we compared the performance of the health-evidence.ca SR search filter to the PH search filter. We then compared the health-evidence.ca SR search filter to other known search filters targeted at capturing systematic reviews in existence at the time (Tables 1, 2 and 3).

**Table 1:** Performance of search terms and filters designed for retrieving systematic reviews in MEDLINE

**Search filter**	Sensitivity*	Specificity**	**Precision**	**Number needed to read**
**health**-**evidence**.**ca**				
health-evidence.ca SR search filter	89.9 (85.0, 93.3)	98.9 (98.9, 98.9)	1.4 (1.3, 1.5)	71.4 (68.7, 75.5)
**Montori**, **et**. **al** (**2005**)				
Sensitive query	99.0 (96.5, 99.7)	62.0 (62.0, 62.0)	0 (0, 0)	2191.2 (2166.3, 2284.3)
‘Balanced query’ (sensitivity > specificity)	99.0 (96.5, 99.7)	87.6 (87.6, 87.6)	0.1 (0.1, 0.1)	712.4 (706.7, 733.4)
Balanced query (specificity > sensitivity)	87.9 (82.8, 91.7)	98.5 (98.5, 98.5)	1.1 (1.0, 1.1)	94.9 (90.9, 100.9)
Specific query	81.6 (75.8, 86.3)	99.3 (99.3, 99.3)	2.0 (1.9, 2.3)	49.4 (46.7, 53.2)
**Shojania and Bero** (**2001**)	85.5 (80.1, 89.7)	99.1 (99.1, 99.1)	1.7 (1.6, 1.8)	57.8 (55.1, 61.8)
**Hunt and McKibbon** (**1997**)4 terms	69.6 (63.0, 75.4)	99.4 (99.4, 99.4)	1.9 (1.7, 2.0)	53.9 (49.7, 59.6)
**Hunt and McKibbon** (**1997**)8 terms	85.5 (80.1, 89.7)	99.2 (99.2, 99.2)	1.9 (1.8, 2.0)	53.4 (50.9, 57.0)
**Boynton**, **et**. **al** (**1998**)				
Sensitivity maximiser	99.5 (97.3, 99.9)	75.6 (75.6, 75.6)	0.1 (0.1, 0.1)	1395.1 (1387.7, 1437.2)
Precision query (> 70%)	47.8 (41.2, 54.6)	99.6 (99.6, 99.6)	2.1 (1.8, 2.5)	46.7 (40.9, 54.4)
**BMJ Clinical Evidence**	88.9 (83.9, 92.5)	99.0 (99.0, 99.0)	1.6 (1.5, 1.7)	61.7 (59.3, 65.5)
**Centre for Reviews and Dissemination**				
For inclusion in DARE	92.8 (88.4, 95.6)	95.7 (95.7, 95.7)	0.4 (0.4, 0.4)	262.2 (254.2, 275.8)
Strategy 1	99.0 (96.5, 99.7)	71.2 (71.2, 71.2)	0.1 (0.1, 0.1)	1693.1 (1659.8, 1773.3)
Strategy 2.1	99.5 (97.3, 99.9)	87.4 (87.4, 87.4)	0.1 (0.1, 0.1)	717.5 (714.2, 736.1)
Strategy 2.2	99.0 (96.5, 99.7)	88.9 (88.9, 88.9)	0.2 (0.2, 0.2)	636.0 (631.0, 654.5)
**Scottish Intercollegiate Guidelines Network Filter**	87.0 (81.7, 90.9)	99.2 (99.2, 99.2)	1.9 (1.8, 2.0)	52.0 (49.7, 55.4)

Values are in percentages (95% confidence intervals).

* Validation data set (n = 207).

** Validation data set (n = 1174817).

Abbreviation: SR – systematic review.

**Table 2:** Performance of search terms and filters designed for retrieving systematic reviews in EMBASE

**Search filter**	Sensitivity*	Specificity**	**Precision**	**Number needed to read**
**health**-**evidence**.**ca**				
health-evidence.ca SR search filter	87.9 (80.3, 92.8)	98.2 (98.2, 98.2)	0.5 (0.5, 0.6)	186.0 (176.0, 208.9)
**Wilcynski and Haynes** (**2007**)				
Sensitive query	96.3 (90.8, 98.5)	72.3 (72.3, 72.3)	0 (0, 0)	2709.5 (2622.5, 2945.2)
‘Small drop in specificity, substantive gain in sensitivity’ query	75.7 (66.7, 82.8)	99.3 (99.3, 99.3)	1.1 (1, 1.2)	88.2 (80.5, 100.1)
Best optimization query	96.3 (90.8, 98.5)	85.5 (85.5, 85.5)	0.1 (0.1, 0.1)	1403.4 (1363.4, 1502.0)
Specific query	63.4 (28.0, 45.9)	99.5 (99.5, 99.5)	0.9 (0.7, 1.1)	117.8 (93.4, 154.2)
**BMJ Clinical Evidence filter**	84.1 (76.0, 89.8)	98.5 (98.5, 98.5)	0.6 (0.5, 0.6)	167.9 (157.0, 186.1)
**Centre for Reviews and Dissemination filter**	66.4 (57.0, 74.6)	97.6 (97.6, 97.6)	0.3 (0.3, 0.3)	341.0 (302.0, 400.0)
**Scottish Intercollegiate Guidelines Network filter**	81.3 (72.9, 87.6)	99.0 (99.0, 99.0)	0.8 (0.8, 0.8)	118.6 (110.1, 132.5)

Values are in percentages (95% confidence intervals).

* Validation data set (n = 107).

** Validation data set (n = 990862).

Abbreviations: SR – systematic review.

**Table 3:** Performance of search terms and filters designed for retrieving systematic reviews in CINAHL

**Search filter**	Sensitivity*	Specificity**	**Precision**	**Number needed to read**
**health**-**evidence**.**ca**				
health-evidence.ca SR search filter	89.9 (93.5, 94.0)	97.6 (97.6, 97.6)	1.8 (1.6, 1.8)	57.2 (54.7, 61.7)
**Wong**, **et**. **al** (**2006**)				
Best sensitivity query	96.1 (91.2, 98.3)	94.6 (94.6, 94.6)	0.8 (0.8, 0.8)	120.8 (118, 127.7)
‘Small drop in sp, substantive gain in sensitivity’ query	45 (36.7, 53.6)	95.3 (95.3, 95.3)	0.5 (0.4, 0.5)	235.3 (193.9, 296.4)
Best optimization (sensitivity > specificity) query	50.4 (42, 58.8)	99.4 (99.4, 99.4)	3.8 (3.2, 4.5)	26.3 (22.5, 31.6)
Best specificity query	47.3 (38.9, 55.8)	99.4 (99.4, 99.4)	3.5 (2.8, 4.1)	29.1 (24.7, 35.5)
**Centre for Reviews and Dissemination Filter**	98.4 (94.5, 99.6)	94.0 (94.0, 94.0)	0.8 (0.7, 0.8)	130.4 (128.9, 136.2)
**McKibbon** (**1998**)	78.3 (70.5, 84.5)	98.9 (98.9, 98.9)	3.2 (2.9, 3.4)	31.7 (29.3, 35.2)

Values are in percentages (95% confidence intervals).

* Validation data set (n = 129).

** Validation data set (n = 272264).

Abbreviations: SR – systematic review.

Our intent was to identify a search filter that resulted in the optimal use of time and resources in updating the health-evidence.ca registry. Specifically, this paper reports the performance of each filter with respect to sensitivity, specificity, precision, and the number needed to read. The best option for our purposes is one that achieves high precision while not compromising sensitivity.

## Methods

The health-evidence.ca SR search filter was evaluated and validated in two distinct ways.

### Health-evidence.ca SR search filter vs. PH search filter

We compared the retrieval performance of the health-evidence.ca SR search filter in MEDLINE, EMBASE, and CINAHL with what we had retrieved using the gold standard, for both our development and validation data sets. The results are reported in Table 4. To test our health-evidence.ca SR search filter, we selected sub-sets from our gold standard for two time periods, the first representing a development (or derivation) data set (January 1 – December 31, 2001) and the second, a validation data set (January 1, 2004 – December 31, 2005). The development data set was used to test and develop the initial health-evidence.ca SR search strategy while the validation data set was used to validate the health-evidence.ca SR search filter.

**Table 4:** **Development and validation data sets for MEDLINE**, **EMBASE**, **and CINAHL**

	**Development** (**2001**)	**Validation** (**2004**–**2005**)
**MEDLINE**		
All articles	503500	1174817
health-evidence.ca SR search filter		
Articles retrieved	4206	13260
Total articles relevant for MEDLINE	53	207
Articles relevant and identified by search filter	46	186
PH search filter		
Articles retrieved	17586	46622
Total articles relevant for MEDLINE	53	207
Articles relevant and identified by search filter	46	191
**EMBASE**		
All articles	990862	453948
health-evidence.ca SR search filter		
Articles retrieved	4105	17443
Total articles relevant for EMBASE	33	107
Articles relevant and identified by search filter	24	94
PH search filter		
Articles retrieved	4663	20919
Total articles relevant for EMBASE	33	107
Articles relevant and identified by search filter	17	68
**CINAHL**		
All articles	96579	272264
health-evidence.ca SR search filter		
Articles retrieved	1895	6619
Total articles relevant for CINAHL	36	129
Articles relevant and identified by search filter	31	116
PH search filter		
Articles retrieved	1443	46630
Total articles relevant for CINAHL	36	129
Articles relevant and identified by search filter	31	114

Abbreviations: SR – systematic review; PH – public health.

### Health-evidence.ca SR search filter vs. other published SR search filters

We evaluated the performance of the health-evidence.ca SR search filter against 28 other known methodological search filters: 15 filters developed for use in MEDLINE, 7 for EMBASE, and 6 for CINAHL. Reference is made to filters that have more than one version (e.g. the Montori filter has four versions) that are discussed independently of each other [30]. The 28 search filters are displayed in Additional Files 1 through 3 (Additional file 1: Table S1 – MEDLINE, Additional file 2: Table S2– EMBASE, and Additional file 3: Table S3 – CINAHL).

Four indices were used to evaluate filter performance: sensitivity, specificity, precision and “number needed to read (NNR)”. Sensitivity is a measure of the proportion of actual positives which are correctly identified. We defined sensitivity as the proportion of systematic reviews identified by the gold standard that were also identified by each search filter. Sensitivity was calculated as:

$$\text{Sensitivity}=\frac{\text{number of systematic reviews retrieved by a search filter}\;}{\text{relevant number of articles in the gold standard}}\text{×}100$$

The higher the sensitivity, the more successful the search filter was in capturing a large number of the articles, in comparison to the gold standard, with 100% meaning there was perfect agreement between the search filter and the gold standard.

Specificity is a measure of the proportion of negatives which are correctly identified. We defined specificity as the proportion of irrelevant articles not retrieved by the search filters. Specificity was calculated as:

$$\text{Specificity}=\frac{\text{number of non}-\text{relevant articles not retrieved by a search filter}\;}{\text{total number of records that are not relevant systematic reviews}}\text{×}\;100$$

Specificity is a reflection of how well a search filter omits non-relevant articles from the retrieved set, which in this case were articles that were not systematic reviews. The specificity score declines if a search filter retrieves an article that it deems to be relevant when, in fact, it is not (a false positive). A specificity of 100% means that the filter recognized all actual non-relevant articles; no articles were retrieved that were not relevant systematic reviews.

Precision (or positive predictive value) is the proportion of retrieved articles that represent relevant articles and can be calculated as:

$$\text{Precision}=\frac{\text{number of relevant records retrieved by a search filter}}{\text{total number of records retrieved by a search filter}}$$

If a search filter has a high degree of precision, it can locate a high number of relevant articles while keeping the number of non-relevant articles retrieved low. A good precision score (N = 1.0) indicates that a high proportion of all articles retrieved for a particular search were actually relevant. In other words, if a search identified 10,000 articles of which 100 were relevant, the precision score would be 0.01, which would be low precision.

Finally, the NNR represents the number of articles that must be read before a relevant article is identified.

$$\text{Number}\;\text{needed}\;\text{to}\;\text{read}=\frac{1}{\text{precision}}$$

For example, if the NNR was 16, then for every 16 articles identified by the search filter and read, one would be deemed relevant.

## Results

Fifty-three relevant articles were identified in the development data set between January 1 and December 31, 2001. Of those 53 relevant reviews, all 53 were published in MEDLINE, 33 in EMBASE and 36 in CINAHL (see Table 4), with some overlap of the same articles being published in more than one of the databases. The initial set of 53 results (development data set) used to test and develop the search strategy was used to explore the sensitivity, specificity, precision, and NNR for both the PH and health-evidence.ca SR search filters.

The second set of 219 results (validation data set), represented a sub-set of the gold standard and was made up of relevant articles indexed in each of the 3 databases of interest between January 1, 2004 and December 31, 2005. Of the 219 articles, 207 were indexed in MEDLINE, 107 in EMBASE, and 129 in CINAHL, again with overlap of the same articles being published in more than one of the databases. During that same time period, a total of 1,174,817 records were indexed in MEDLINE, 990,862 records in EMBASE, and 272,264 records in CINAHL (see Table 4). Table 5 displays the performance of the health-evidence.ca SR search filter in comparison to the PH search filter for sensitivity, specificity, precision and NNR. A comparison of results for the validation data set to results for the development data set demonstrated the same trend: although the sensitivity of the health-evidence.ca SR and PH search filters was comparable (89.9% vs. 92.3%), the health-evidence.ca SR search filter was more precise (1.4 vs. 0.4) and offered a lower NNR (71.4 vs. 244.9). Results of the comparison on each individual criterion are reported in Table 5.

**Table 5:** **Performance of the health**-**evidence**.**ca SR search filter compared to the PH search filter in retrieving systematic reviews in MEDLINE**, **EMBASE**, **CINAHL**

**MEDLINE**				
**Search filter**	**Sensitivity**	**Specificity**	**Precision**	**Number needed to read**
health-evidence.ca SR search filter†				
Development	86.8 (75.2, 93.5)	99.2 (99.2, 99.2)	1.1 (0.9, 1.2)	91.6 (85.0, 105.9)
Validation	89.9 (85.0, 93.3)	98.9 (98.9, 98.9)	1.4 (1.3, 1.5)	71.4 (68.7, 75.5)
PH search filter‡				
Development	86.8 (75.2, 93.5)	96.5 (96.5, 96.5)	0.3 (0.2, 0.3)	384.4 (356.0, 446.8)
Validation	92.3 (87.8, 95.2)	96.0 (96.0, 96.0)	0.4 (0.4, 0.4)	244.9 (237.1, 258.0)
**EMBASE**				
**Search filter**	**Sensitivity**	**Specificity**	**Precision**	**Number Needed to Read**
health-evidence.ca SR search filter				
Development	72.7 (55.8, 84.9)	99.1 (99.1, 99.1)	0.6 (0.4, 0.7)	171.6 (146.7, 224.6)
Validation	87.9 (80.2, 92.8)	98.2 (98.2, 98.2)	0.5 (0.5, 0.6)	186.0 (176.0, 208.9)
PH search filter				
Development	48.5 (32.5, 64.8)	99.0 (99.0, 99.0)	0.3 (0.2, 0.5)	294.7 (219.4, 444.1)
Validation	63.6 (54.1, 72.0)	97.9 (97.9, 97.9)	0.3 (0.3, 0.4)	311.5 (273.6, 368.0)
**CINAHL**				
**Search filter**	**Sensitivity**	**Specificity**	**Precision**	**Number Needed to Read**
health-evidence.ca SR search filter				
Development	86.1 (71.4, 93.9)	98.1 (98.1, 98.1)	1.6 (1.4, 1.8)	61.3 (56.1, 74.3)
Validation	89.9 (93.5, 94.0)	97.6 (97.6, 97.6)	1.8 (1.6, 1.8)	57.2 (54.7, 61.7)
PH search filter				
Development	86.1 (71.4, 93.9)	98.5 (98.5, 98.5)	2.1 (1.8, 2.3)	46.7 (42.7, 56.5)
Validation	37.2 (29.4, 45.8)	98.2 (98.2, 98.2)	1.0 (0.8, 1.2)	107.8 (86.8, 138.6)

Values are in percentages (95% confidence intervals).

†, ‡: see additional files for full search strategy.

Abbreviations: SR – systematic review; PH – public health.

### Comparison of the health-evidence.ca SR search filter to the PH search filter

#### Sensitivity

Both the health-evidence.ca SR search filter and the PH search filter returned a high yield of articles in all three databases. The health-evidence.ca SR search filter identified 13,260 articles in MEDLINE, which included 186 of the 207 relevant articles identified in the gold standard, resulting in a sensitivity score of 89.9%. The PH search filter identified 46,622 articles in MEDLINE, capturing 191 of the 207 gold standard articles, representing a slightly higher sensitivity score of 92.3%. In EMBASE and CINAHL, the health-evidence.ca SR search filter outperformed the PH search filter, scoring 87.4% vs. 63.6% in EMBASE and 89.9% vs. 37.2% in CINAHL. Furthermore, in EMBASE the health-evidence.ca SR search filter retrieved 94 of 107 gold standard results whereas the PH search strategy retrieved 68 of 107. In CINAHL, the health-evidence.ca SR search filter retrieved 116 of 129 gold standard results while the PH strategy retrieved 114 of 129 gold standard results.

#### Specificity

In addition to being sensitive, the health-evidence.ca SR search filter demonstrated a slightly higher degree of specificity than the PH search filter in MEDLINE (98.9% vs. 96.0%). The health-evidence.ca SR and PH search filters performed comparably in EMBASE (98.2% vs. 97.9%) and CINAHL (97.6% vs. 98.2%).

#### Precision and number needed to read (NNR)

There was an almost four-fold difference between the precision scores of the health-evidence.ca SR and PH search filters in MEDLINE (1.4 vs. 0.4), representing a substantial reduction in the number of irrelevant articles needing to be read. The NNR in MEDLINE differed greatly from 71.4 articles for the health-evidence.ca SR search filter to 244.9 for the PH search filter. In EMBASE, precision was only slightly better for the health-evidence.ca SR search filter (SR: 0.6 vs. PH: 0.3) as well as the NNR (SR: 186 vs. PH: 244.9). Despite retrieving a higher number of articles from CINAHL, the health-evidence.ca SR search filter had higher precision (1.8 vs. 1.0) and performed better on NNR (57.2 vs. 107.8).

### Comparison of the health-evidence.ca SR search filter to other published SR search filters

#### MEDLINE

Table 1 displays the performance of the health-evidence.ca SR search filter in comparison to the 15 identified search filters used by others in MEDLINE for sensitivity, specificity, precision and NNR.

##### Sensitivity

Five of the search filters had sensitivity scores greater than 90%, with the health-evidence.ca SR search filter obtaining slightly less at 89.9%. All of the searches obtained a sensitivity level of 80% or greater with the exception of the Hunt & McKibbon (1997) hedge and the Boynton et al. (1998) precision query.

##### Specificity

All but three filters achieved a level of specificity above 85%. Most of these high specificity scores accompanied a high degree of sensitivity except Hunt & McKibbon (1997) and the Boynton et al. precision query (1998), which performed better in eliminating non-relevant papers than in selecting relevant papers from MEDLINE. With a specificity of 98.9%, the health-evidence.ca SR search filter was outperformed by a small margin by six of the filters [30,31,35-37].

##### Precision

The most precise search filter (Boynton et al. precision query) demonstrated the highest number of relevant articles returned as a proportion of the entire results set (2.1). A number of other filters performed within close range of the Boynton et al. precision query (1.6-2.0), with the health-evidence.ca SR search filter having slightly less precision at 1.4.

##### Number needed to read

The Boynton et al. precision query had the lowest NNR at 50, but at the expense of sensitivity, which was only 47.8%. The health-evidence.ca SR search filter and the BMJ Clinical Evidence filter performed the best on NNR at 71.4 and 61.7 respectively, while maintaining a high level of sensitivity (>85%).

The health-evidence.ca SR search filter performed well in MEDLINE in terms of overall balance of sensitivity (89.9%), specificity (98.9%), precision (1.4), and NNR (71.4). The health-evidence.ca SR search filter along with five other filters [30,31,35-37] offered relatively high sensitivity (85.5%-88.9%) combined with good performance on specificity (98.5%-99.2%), precision (1.1-1.9), and number needed to read (52.0-94.9).

#### EMBASE

Table 2 describes the results of the health-evidence.ca SR search filter in comparison to the seven other search filters tested in EMBASE. The health-evidence.ca SR and Scottish Intercollegiate Guidelines Network [37] search filters performed the best overall in terms of the combination of outcomes for sensitivity (87.9% and 81.3%), specificity (98.2% and 99.0%), precision (0.5 and 0.8) and NNR (186.0 and 118.6). The health-evidence.ca SR search filter, while having greater sensitivity, resulted in an additional 67 articles having to be read in comparison to the Scottish Intercollegiate Guidelines Network filter.

##### Sensitivity

The health-evidence.ca SR search filter’s sensitivity of 87.9% was slightly lower than that of the two top performing search filters which both obtained sensitivity scores of 96.3% (Wilcynski and Haynes, Sensitive query; Wilcynski and Haynes, Best optimization query).

##### Specificity

All but the Wilcynski and Haynes (2007) search filter (sensitive query) achieved a level of specificity above 85%, with the health-evidence.ca SR search filter achieving 98.2%. The health-evidence.ca SR search filter was outperformed by the two Wilcynski and Haynes filters (99.3% for the ‘Small drop in specificity, substantive gain in sensitivity’ query, and 99.5% for the specific query), the BMJ Best Clinical Evidence filter (98.5%), and the Scottish Intercollegiate Guidelines Network filter (99.0%).

##### Precision

The most precise filter had a score of 1.1 (Wilcynski and Haynes, ‘Small drop in specificity, substantive gain in sensitivity’ query) while retaining a high level of sensitivity (75.7%). The health-evidence.ca SR search filter offered moderate precision (0.5) in comparison.

##### Number needed to read

The best performing filters for NNR were SIGN, BMJ Clinical Evidence filter, and the health-evidence.ca SR search filter at 118, 167.9, and 186 respectfully. Although the Wilcynski and Haynes (‘Small drop in specificity, substantive gain in sensitivity’ query) filter offered an NNR of 88.2, its sensitivity was much lower than that of other filters at 75.7%.

#### CINAHL

Table 3 presents the results of the health-evidence.ca SR search filter along with the six other search filters tested in CINAHL. Although not performing with the best result on any single outcome, the health-evidence.ca SR search filter appeared to offer the best overall combination of sensitivity (89.9%), specificity (97.6%), precision (1.8), and NNR (57.2).

##### Sensitivity

Two search strategies achieved a sensitivity of greater than 95% (Wong, Best sensitivity; Centre for Reviews and Dissemination (CRD) [38] filters), with the health-evidence.ca SR search filter achieving 89.9% sensitivity.

##### Specificity

The Wong Best sensitivity query scored highest on specificity (99.4%), matched by the Wong Best optimization (sensitivity > specificity) query (99.4%). The Wong queries were followed closely in specificity by the McKibbon (1998) filter (98.9%) and the health-evidence.ca SR search filter (97.6%).

##### Precision

The most precise search filter was Wong’s, Best optimization query at 3.8, followed by the Best Specificity Query [33] at 3.5, McKibbon [39] at 3.2, and the health-evidence.ca SR search filter at 1.8.

##### Number needed to read

Though sensitivity for the health-evidence.ca SR search filter (89.9%) was slightly lower than The Wong Best sensitivity query (96.1%) and CRD filter (98.4%), those two filters produced an NNR of 120.8 and 130.4, respectively, while the NNR for the health-evidence.ca SR search filter was only 57.2.

## Discussion

The objective of health-evidence.ca is to contribute to evidence-informed decision making in public health by facilitating access to published systematic reviews evaluating the effectiveness of public health and health promotion interventions. An optimal search filter for health-evidence.ca is one that has high sensitivity, specificity, and precision and a relatively low NNR. However, any reduction in NNR was desirable. A filter such as this allows us to have confidence that all relevant articles will be identified (sensitivity), fewer non-relevant articles will be retrieved (specificity), most of the identified articles will be relevant (precision), and the NNR will be reduced. Reducing the NNR is of great importance since screening is a resource- and time-intensive process.

Although a search filter may perform exceptionally well on any single outcome, it is the balance of performance across these four domains – sensitivity, specificity, precision, NNR – that distinguishes the best filter for our purposes. By replacing the PH search filter with the health-evidence.ca SR search filter, the overall number of articles retrieved from health-evidence.ca electronic searches was greatly reduced without losing relevant content. The balance struck by the SR search filter means that this filter would be useful to those wishing to retrieve systematic reviews related to health care, with wider application than that of our own database of reviews on the effectiveness of interventions. The desired benefit of filters is that they save time both in search strategy development and screening. One study demonstrated how filters reduce the number of results needed to screen [37], while another found that saving time both in search strategy development and screening of results was the most common benefit reported by librarians [38]. For our purposes, the health-evidence.ca SR search filter offered overall improvements in specificity and precision, with the associated decrease in the NNR, substantially decreasing screening time. The desired improvement in precision was feasible while only minimally impacting the sensitivity of the search strategy. The results of this study illustrate that for the most part, the health-evidence.ca SR search filter outperformed the PH search filter with respect to sensitivity, specificity, precision and NNR in all three databases. However, it was the overall balance among these variables and the fact that high precision could be combined with high sensitivity that made the health-evidence.ca SR search filter the optimal choice for identifying systematic reviews evaluating the effectiveness of interventions.

When compared to other filters in MEDLINE, EMBASE and CINAHL, overall, the health-evidence.ca SR search filter offered the right balance of sensitivity, specificity, precision, and NNR. Although other filters had higher sensitivity scores than the health-evidence.ca SR search filter in MEDLINE, these higher sensitivity scores were generally accompanied by poorer precision and NNR performance. In EMBASE, the health-evidence.ca SR and Scottish Intercollegiate Guidelines Network search filters performed the best overall and were comparable in terms of performance across all of the outcome measures. Likewise in CINAHL, though the health-evidence.ca SR search filter did not outperform other filters on any single outcome, it offered the most robust overall result of high sensitivity and specificity with a reasonably low NNR in comparison to other filters.

The health-evidence.ca SR search filter streamlines the process of locating and screening relevant reviews by allowing us to effectively search health databases with a simpler strategy that maintains a high level of both sensitivity and precision. The task of searching the health databases for every relevant systematic review evaluating effectiveness of public health interventions is a challenging one that requires balance. Because of the growth of the literature in the area of systematic reviews, highly sensitive searches often come up with result sets that are unmanageably large. However, if a search is too specific, then it has the risk of missing relevant articles. It is important to establish the right balance in the trade-off between sensitivity and specificity depending on what will best serve the purpose at hand [39,40]. Using the health-evidence.ca SR search filter has allowed us to achieve the right balance in our searches by retaining greater than 85% sensitivity across all three databases, while reducing the NNR by two thirds. We estimate that this has translated into a savings of 384 hours of staff time per quarterly update of health-evidence.ca by reducing the hours required to execute database searches, screen results, retrieve full-text versions of potentially relevant reviews, and test reviews for relevance. The reduction has meant that resources are available for the exploration and development of new protocols for searching other relevant but previously unexplored electronic databases covering areas such as environmental health, social welfare, and veterinary sciences for relevant public health content.

The health-evidence.ca SR search filter is an easy-to-use tool. It can be entered into the OVID interface for searching in MEDLINE and EMBASE. Compared to other more complex filters, the health-evidence.ca SR search filter is easily entered. A survey of librarians revealed that users find search strings too long [38,40]. The SR search filter used by health-evidence.ca is a relatively short search filter, with other authors also finding that the brief search filters work well. Our results, which are similar to those of others [39,38], indicate that methodological search filters can be as or more effective than content filters for retrieving relevant systematic reviews [27-35,39]. Using a methodological filter allows us to circumvent the need to generate an accurate and all encompassing definition of public health that can be translated and applied across indexing systems within different databases. However, if desired, the search strategy can be combined (using Boolean logic, e.g. AND) with topic-specific search terms to reduce the number of articles retrieved, if applied for a specific topic area (e.g. influenza).

### Limitations

Searching was conducted in OVID’s search interface for all three databases; other search interfaces for these databases (e.g. PubMed) may handle the searches somewhat differently. As of August 30, 2008, CINAHL moved from OVID Technologies to be hosted by EBSCO, exclusively. Unfortunately, this change to EBSCO renders the CINAHL filters included in this paper, including our filters, out of date. The performance of these filters would require reevaluating them in the EBSCO platform before their application. This brings light to a key limitation of search filters – creation dates must always be considered before using a filter as changes to indexing terms and hosting platforms can impact filter function.

The sensitivity scores calculated for each search filter can be applied to broader searches for systematic reviews evaluating various interventions and are not necessarily applicable only to public health interventions. However, precision and NNR scores were calculated specifically for public health content and cannot be generalized to topic areas outside of public health. The low precision scores yielded across all search filters were expected, since precision is generally low when searching large databases [39,40]. Lastly, our group’s own manual screening set was used as the gold standard. Although a consistent set of relevance criteria were applied to generate this results set, screening was shared between two authors (MD, KD), and several other members of the health-evidence.ca team. Although either MD or KD acted as second reviewer on each article, there was still potential for reviewer bias through the involvement of a small number of reviewers. Additionally, having a combination of both systematic review methodology indexing terms and public health indexing terms in our PH search filter dually limited our results sets, retrieving only content which met all requirements for both methodology and public health content.

## Conclusions

Methodological search filters may reduce the number of articles needed to be screened and read while maintaining a high level of sensitivity for finding relevant articles. The health-evidence.ca SR search filter is a simpler, yet effective tool to retrieve systematic reviews evaluating the effectiveness of interventions across MEDLINE, EMBASE, and CINAHL. Our findings support the use of methodological search filters for retrieving systematic reviews [27-34,39]. These filters save considerable screening time, which translates into a quicker turnaround for relevant reviews to be published in health-evidence.ca

## Abbreviations

SR: Systematic Review; KTE: Knowledge Translation and Exchange; PH: Public Health; NNR: Number Needed to Read.

## Competing interests

The authors declare that they have no competing interests.

## Authors’ contributions

EL conceived of the study, participated in the analysis and wrote the first draft of the manuscript. KD and MD completed subsequent drafts and the final version of the manuscript. LM, DT, and HH consulted on the analysis. All authors read and approved the final manuscript.

## Pre-publication history

The pre-publication history for this paper can be accessed here:

http://www.biomedcentral.com/1471-2288/12/51/prepub

## Supplementary Material

Additional file 1 **Table S1.** MEDLINE systematic review filters tested, in order of highest to lowest sensitivity. Click here for file

Additional file 2 **Table S2.** EMBASE systematic review filters tested, in order of highest to lowest sensitivity. Click here for file

Additional file 3 **Table S3.** CINAHL systematic review filters tested, in order of highest to lowest sensitivity. Click here for file
