# Supplementary material for: An optimal search filter for retrieving systematic reviews and meta-analyses
Source: BMC Med Res Methodol. 2012 Apr 18;12:51. doi: 10.1186/1471-2288-12-51 (PMC3515398; doi:10.1186/1471-2288-12-51)
Supplement: Additional file 1 — Table S1. MEDLINE systematic review filters tested, in order of highest to lowest sensitivity. [file 1471-2288-12-51-S1.doc]

# Additional files

**Additional file 1 – MEDLINE systematic review filters tested, in order of highest to lowest sensitivity**

| **Filter Name** | **Filter Strategy**  **(OVID Technologies Format)** | **Performance**  Values are in percentages (95% confidence intervals)  * Development dataset (n=82), Validation dataset (n=207)  ** Development dataset (n=503500), Validation dataset (n=1174817) | | | |
| --- | --- | --- | --- | --- | --- |
|  |  | ***Sensitivity*** | ***Specificity*** | ***Precision*** | ***Number Needed to Read (NNR)*** |
| Boynton (Sensitivity maximiser) | 1. meta.ab.  2. synthesis.ab.  3. literature.ab.  4. randomized.hw.  5. published.ab.  6. meta-analysis.pt.  7. extraction.ab.  8. trials.hw.  9. controlled.hw.  10. search.ab.  11. MEDLINE.ab.  12. selection.ab.  13. sources.ab.  14. trials.ab.  15. review.ab.  16. review.pt.  17. articles.ab.  18. reviewed.ab.  19. english.ab.  20. language.ab.  21. or/1-20 | 99.5 (97.3, 99.9) | 75.6 (75.6, 75.6) | 0.1 (0.1, 0.1) | 1395.1  (1387.7, 1437.2) |
| Centre for Reviews and Dissemination (Strategy 2.1) | 1. review.ab.  2. review.pt.  3. meta-analysis.ab.  4. meta-analysis.pt.  5. meta-analysis.ti.  6. or/1-5  7. letter.pt.  8. comment.pt.  9. editorial.pt.  10. or/7-9  11. 6 not 10 | 99.5 (97.3, 99.9) | 87.4 (87.4, 87.4) | 0.1 (0.1, 0.1) | 717.5  (714.2, 736.1) |
| Montori (Sensitive query) | 1. search:.tw.  2. meta-analysis.mp,pt.  3. review.pt.  4. di.xs.  5. associated.tw.  6. or/1-5 | 99.0 (96.5, 99.7) | 62.0 (62.0, 62.0) | 0 (0, 0) | 2191.2  (2166.3, 2284.3) |
| Montori (Balanced query, sn>sp) | 1. meta-analysis.mp,pt.  2. review.pt.  3. search:.tw.  4. or/1-4 | 99.0 (96.5, 99.7) | 87.6 (87.6, 87.6) | 0.1 (0.1, 0.1) | 712.4  (706.7, 733.4) |
| Centre for Reviews and Dissemination (Strategy 1) | 1. review.ab.  2. review.pt.  3. meta-analysis.ab.  4. meta-analysis.pt.  5. meta-analysis.ti.  6. or/1-5  7. letter.pt.  8. comment.pt.  9. editorial.pt.  10. or/7-9  11. 6 not 10 | 99.0 (96.5, 99.7) | 71.2 (71.2, 71.2) | 0.1 (0.1, 0.1) | 1693.1  (1659.8, 1773.3) |
| Centre for Reviews and Dissemination (Strategy 2.2) | 1. meta-analysis/  2. review literature/  3. meta-analy$.tw.  4. metaanal$.tw.  5. (systematic$ adj4 (review$ or overview$))  6. meta-analysis.pt.  7. review.pt.  8. review.ti.  9. review literature.pt.  10. or/1-9  11. case report/  12. letter.pt.  13. historical article.pt.  14. review of reported cases.pt.  15. review,multicase.pt.  16. or/11-15  17. 10 not 16 | 99.0 (96.5, 99.7) | 88.9 (88.9, 88.9) | 0.2 (0.2, 0.2) | 636.0  (631.0, 654.5) |
| Centre for Reviews and Dissemination: For inclusion in DARE | 1. controlled.ab.  2. design.ab.  3. evidence.ab.  4. extraction.ab.  5. randomized controlled trials/  6. meta-analysis.pt.  7. review.pt.  8. sources.ab.  9. studies.ab.  10. or/1-9  11. letter.pt.  12. comment.pt.  13. editorial.pt.  14. or/11-13  15. 10 not 14 | 92.8 (88.4, 95.6) | 95.7 (95.7, 95.7) | 0.4 (0.4, 0.4) | 262.2  (254.2, 275.8) |
| health-evidence.ca: Public health content filter | 1. exp health promotion/  2. exp health education/  3. exp primary prevention/  4. exp preventive health services/  5. exp education/  6. prevention.mp.  7. exp community health services/  8. exp public health/  9. or/1-8  10. (systematic.mp OR meta analysis/ OR review/)  11. 9 and 10 | 92.3 (87.8, 95.2) | 96.0 (96.0, 96.0) | 0.4 (0.4, 0.4) | 244.9  (237.1, 258) |
| health-evidence.ca: Systematic review methodology filter | 1. MEDLINE.tw.  2. systematic review.tw.  3. meta-analysis.pt.  4. intervention$.ti  5. or/1-4 | 89.9 (85.0, 93.3) | 98.9 (98.9, 98.9) | 1.4 (1.3, 1.5) | 71.4  (68.7, 75.5) |
| BMJ Clinical Evidence | 1. ("review" or "review academic" or "review tutorial").pt.  2. (MEDLINE or medlars or embase or pubmed).tw,sh.  3. (scisearch or psychinfo or psycinfo).tw,sh.  4. (psychlit or psyclit).tw,sh.  5. cinahl.tw,sh.  6. ((hand adj2 search$) or (manual$ adj2 search$)).tw,sh.  7. (electronic database$ or bibliographic database$ or computeri?ed database$ or online database$).tw,sh.  8. (pooling or pooled or mantel haenszel).tw,sh.  9. (retraction of publication or retracted publication).pt.  10. (peto or dersimonian or der simonian or fixed effect).tw,sh.  11. or/2-10  12. 1 and 11  13. meta-analysis.pt.  14. meta-analysis.sh.  15. (meta-analys$ or meta analys$ or metaanalys$).tw,sh.  16. (systematic$ adj5 review$).tw,sh.  17. (systematic$ adj5 overview$).tw,sh.  18. (quantitativ$ adj5 review$).tw,sh.  19. (quantitativ$ adj5 overview$).tw,sh.  20. (quantitativ$ adj5 synthesis$).tw,sh.  21. (methodologic$ adj5 review$).tw,sh.  22. (methodologic$ adj5 overview$).tw,sh.  23. (integrative research review$ or research integration).tw.  24. or/13-23  25. 12 or 24 | 88.9 (83.9, 92.5) | 99.0 (99.0, 99.0) | 1.6 (1.5, 1.7) | 61.7  (59.3, 65.5) |
| Montori (Balanced query, sp>sn) | 1. Cochrane database of systematic reviews.jn.  2. search:.tw.  3. meta-analysis.pt.  4. MEDLINE.tw.  5. systematic review.tw.  6. or/1-6 | 87.9 (82.8, 91.7) | 98.5 (98.5, 98.5) | 1.1 (1.0, 1.1) | 94.9  (90.9, 100.9) |
| Scottish Intercollegiate Guidelines Network | 1. Meta-Analysis as Topic/  2. meta analy$.tw.  3. metaanaly$.tw.  4. Meta-Analysis/  5. (systematic adj (review$1 or overview$1)).tw.  6. exp Review Literature as Topic/  7. or/1-6  8. cochrane.ab.  9. embase.ab.  10. (psychlit or psyclit).ab.  11. (psychinfo or psycinfo).ab.  12. (cinahl or cinhal).ab.  13. science citation index.ab.  14. bids.ab.  15. cancerlit.ab.  16. or/8-15  17. reference list$.ab.  18. bibliograph$.ab.  19. hand-search$.ab.  20. relevant journals.ab.  21. manual search$.ab.  22. or/17-21  23. selection criteria.ab.  24. data extraction.ab.  25. 23 or 24  26. Review/  27. 25 and 26  28. Comment/  29. Letter/  30. Editorial/  31. animal/  32. human/  33. 31 not (31 and 32)  34. or/28-30,33  35. 7 or 16 or 22 or 27  36. 35 not 34 | 87.0 (81.7, 90.9) | 99.2 (99.2, 99.2) | 1.9 (1.8, 2.0) | 52.0  (49.7, 55.4) |
| Shojania and Bero | 1. meta-analysis.pt. or meta-analysis.tw. or metanalysis.tw.  2. Cochrane.tw or MEDLINE.tw. or CINAHL.tw. or (National.tw. and Library.tw.)  3. (handsearch*.tw. or search*.tw.  or searching.tw. and (hand.tw.  or manual.tw. or electronic.tw.  or bibliographi*.tw. or database*  or #2)  4. (review.pt. or guideline.pt. or  consensus.ti. or guideline*.ti. or literature.ti. or overview.ti. or review.ti.) and (#2 or #3)  5. (synthesis.ti. or overview.ti. or review.ti. or survey.ti.) and  (systematic.ti. or critical.ti. or methodologic.ti. or quantitative.ti. or qualitative.ti. or literature.ti. or evidence.ti. or evidencebased.ti.)  6. (#1 or #4 or #5) not (case*.ti.  or report.ti. or editorial.pt. OR or comment.pt. or letter.pt.) | 85.5 (80.1, 89.7) | 99.1 (99.1, 99.1) | 1.7 (1.6, 1.8) | 57.8  (55.1, 61.8) |
| Hunt and McKibbon (Sensitive) | 1. meta-analysis.pt  2. meta-anal:.tw.  3. meta-anal:.tw.  4. quantitative: review:.tw. or quantitative: overview:.tw.  5. systematic: review:.tw. or systematic: overview:.tw.  6. methodologic: review:.tw. or methodologic: overview:.tw.  7. review.pt. and MEDLINE.tw.  8. or/1-7 | 85.5 (80.1, 89.7) | 99.2 (99.2, 99.2) | 1.9 (1.8, 2.0) | 53.4  (50.9, 57.0) |
| Montori (Specific) | 1. MEDLINE.tw.  2. systematic review.tw.  3. meta-analysis.pt  4. or/1-3 | 81.6 (75.8, 86.3) | 99.3 (99.3, 99.3) | 2.0 (1.9, 2.3) | 49.4  (46.7, 53.2) |
| Hunt and McKibbon (Simple) | 1. meta-analysis.pt  2. meta-anal:.tw.  3. review.pt and MEDLINE.tw.  4. or/1-3 | 69.6 (63.0, 75.4) | 99.4 (99.4, 99.4) | 1.9 (1.7, 2.0) | 53.9  (49.7, 59.6) |
| Boynton (Precision > 70%) | 1. MEDLINE.ab | 47.8 (41.2, 54.6) | 99.6 (99.6, 99.6) | 2.1 (1.8, 2.5) | 46.7  (40.9, 54.4) |
