# Supplementary material for: An optimal search filter for retrieving systematic reviews and meta-analyses
Source: BMC Med Res Methodol. 2012 Apr 18;12:51. doi: 10.1186/1471-2288-12-51 (PMC3515398; doi:10.1186/1471-2288-12-51)
Supplement: Additional file 2 — Table S2. EMBASE systematic review filters tested, in order of highest to lowest sensitivity. [file 1471-2288-12-51-S2.doc]

# Additional files

**Additional file 2 – EMBASE systematic review filters tested, in order of highest to lowest sensitivity**

| **Filter Name** | **Filter Strategy**  **(OVID Technologies Format)** | **Performance**  Values are in percentages (95% confidence intervals)  * Validation dataset (n=107)  ** Validation dataset (n=990862) | | | |
| --- | --- | --- | --- | --- | --- |
|  |  | ***Sensitivity*** | ***Specificity*** | ***Precision*** | ***Number Needed to Read (NNR)*** |
| Wilcynski and Haynes  Sensitive Query | 1. exp methodology/  2. search:.tw.  3. review.pt.  4. or/1-3 | 96.3 (90.8, 98.5) | 72.3 (72.3, 72.3) | 0 (0, 0) | 2709.5  (2622.5, 2945.2) |
| Wilcynski and Haynes  Best optimization, sn>sp | 1. meta-analy:.mp.  2. search:.tw.  3. review.pt.  4. or/1-3 | 96.3 (90.8, 98.5) | 85.5 (85.5, 85.5) | 0.1 (0.1, 0.1) | 1403.4  (1363.4, 1502.0) |
| health-evidence.ca Systematic review filter | 1. MEDLINE.tw.  2. exp systematic review/ or systematic review.tw  3. meta-analysis/  4. intervention$.ti  5. or/1-4 | 87.9 (80.3, 92.8) | 98.2 (98.2, 98.2) | 0.5 (0.5, 0.6) | 186.0  (176.0, 208.9) |
| BMJ Clinical Evidence | 1. exp review/  2. (MEDLINE or medlars or embase or pubmed).ti,ab,sh.  3. (scisearch or psychlit or psyclit).ti,ab,sh.  4. (psycinfo or psychinfo).ti,ab,sh.  5. cinahl.ti,ab,sh.  6. ((hand adj2 search$) or (manual$ adj search$)).tw.  7. ((electronic adj database$) or (bibliographic adj database$)).tw.  8. ((pooled adj analys$) or pooling).tw.  9. (peto or dersimonian or (fixed adj effect) or mantel haenszel).tw.  10. RETRACTED ARTICLE/  11. or/2-10  12. 1 and 11  13. exp meta analysis/  14. meta?analys$.tw,sh.  15. (systematic$ adj5 review$).tw,sh.  16. (systematic$ adj5 overview$).tw,sh.  17. (quantitativ$ adj5 review$).tw,sh.  18. (quantitativ$ adj5 overview$).tw,sh.  19. (methodologic$ adj5 review$).tw,sh.  20. (methodologic$ adj5 overview$).tw,sh.  21. ((integrative adj5 research adj5 review$) or (research adj5 integration)).tw.  22. (quantitativ$ adj5 synthesi$).tw,sh.  23. or/13-22  24. 12 or 23 | 84.1 (76.0, 89.8) | 98.5 (98.5, 98.5) | 0.6 (0.5, 0.6) | 167.9  (157.0, 186.1) |
| Scottish Intercollegiate Guidelines Network | 1. Meta-Analysis as Topic/  2. meta analy$.tw.  3. metaanaly$.tw.  4. Meta-Analysis/  5. (systematic adj (review$1 or overview$1)).tw.  6. exp Review Literature as Topic/  7. or/1-6  8. cochrane.ab.  9. embase.ab.  10. (psychlit or psyclit).ab.  11. (psychinfo or psycinfo).ab.  12. (cinahl or cinhal).ab.  13. science citation index.ab.  14. bids.ab.  15. cancerlit.ab.  16. or/8-15  17. reference list$.ab.  18. bibliograph$.ab.  19. hand-search$.ab.  20. relevant journals.ab.  21. manual search$.ab.  22. or/17-21  23. selection criteria.ab.  24. data extraction.ab.  25. 23 or 24  26. Review/  27. 25 and 26  28. Comment/  29. Letter/  30. Editorial/  31. animal/  32. human/  33. 31 not (31 and 32)  34. or/28-30,33  35. 7 or 16 or 22 or 27  36. 35 not 34 | 81.3 (72.9, 87.6) | 99.0 (99.0, 99.0) | 0.8 (0.8, 0.8) | 118.6  (110.1, 132.5) |
| Wilcynski and Haynes  “Small drop in specificity, substantive gain in sensitivity” Query | 1. meta-analysis.tw.  2. systematic review.tw.  3. MEDLINE.tw.  4. or/1-3 | 75.7 (66.7, 82.8) | 99.3 (99.3, 99.3) | 1.1 (1, 1.2) | 88.2  (80.5, 100.1) |
| Centre for Reviews and Dissemination (for inclusion in DARE) | 1. exp meta analysis/  2. meta-analys$.ti,ab.  3. metaanalys$.ti,ab.  4. meta analys$.ti,ab.  5. review$.ti.  6. overview$.ti.  7. (synthes$ adj3 (literature$ or research$ or studies or data)).ti,ab.  8. pooled analys$.ti,ab.  9. ((data adj2 pool$) and studies).mp.  10. (MEDLINE or medlars or embase or cinahl or scisearch or psychinfo or psycinfo or psychlit or  psyclit).ti,ab.  11. ((hand or manual or database$ or computer$) adj2 search$).ti,ab.  12. ((electronic or bibliographic$) adj2 (database$ or data base$)).ti,ab.  13. ((review$ or overview$) adj10 (systematic$ or methodologic$ or quantitativ$ or research$ or  literature$ or studies or trial$ or effective$)).ab.  14. 1 or 2 or 3 or 4 or 5 or 6 or 7 or 8 or 9 or 10 or 11 or 12 or 13  15. (retrospective$ adj2 review$).ti,ab,sh.  16. (case$ adj2 review$).ti,ab,sh.  17. (record$ adj2 review$).ti,ab,sh.  18. (patient$ adj2 review$).ti,ab,sh.  19. (patient$ adj2 chart$).ti,ab,sh.  20. (peer adj2 review$).ti,ab,sh.  21. (chart$ adj2 review$).ti,ab,sh.  22. (case$ adj2 report$).ti,ab,sh.  23. (rat or rats or mouse or mice or hamster or hamsters or animal or animals or dog or dogs or  cat or cats or bovine or sheep).ti,ab,sh.  24. 15 or 16 or 17 or 18 or 19 or 20 or 21 or 22 or 23  25. 14 not 24  26. editorial.pt.  27. letter.pt.  28. 26 or 27  29. 25 not 28  30. exp animal/  31. exp nonhuman/  32. 30 or 31  33. exp human/  34. 32 not (32 and 33)  35. 29 not 34 | 66.4 (57.0, 74.6) | 97.6 (97.6, 97.6) | 0.3 (0.3, 0.3) | 341.0  (302.0, 400.0) |
| health-evidence.ca Public Health filter | 1. exp health promotion/  2. exp health education/  3. exp primary prevention/  4. exp preventive health services/  5. exp education/  6. exp public health/  7. exp prevention/  8. exp community care/  9. exp community medicine/  10. or/1-9  11. systematic review.mp.  12. meta analysis/  13. review/  14. or/ 11-13  15. 10 and 15 | 63.6 (54.1, 72.0) | 97.9 (97.9, 97.9) | 0.3 (0.3, 0.4) | 311.5  (273.6, 368) |
| Wilcynski and Haynes  Specific Query | 1. meta-analysis.tw.  2. systematic review.tw.  3. or/1-2 | 63.4 (28.0, 45.9) | 99.5 (99.5, 99.5) | 0.9 (0.7, 1.1) | 117.8  (93.4, 154.2) |
