# Supplementary material for: An optimal search filter for retrieving systematic reviews and meta-analyses
Source: BMC Med Res Methodol. 2012 Apr 18;12:51. doi: 10.1186/1471-2288-12-51 (PMC3515398; doi:10.1186/1471-2288-12-51)
Supplement: Additional file 3 — Table S3. CINAHL systematic review filters tested, in order of highest to lowest sensitivity. [file 1471-2288-12-51-S3.doc]

# Additional files

**Additional file 3 – CINAHL systematic review filters tested, in order of highest to lowest sensitivity**

| **Filter Name** | **Filter Strategy**  **(OVID Technologies Format)** | **Performance**  Values are in percentages (95% confidence intervals)  * Validation dataset (n=129)  ** Validation dataset (n=272264) | | | |
| --- | --- | --- | --- | --- | --- |
|  |  | ***Sensitivity*** | ***Specificity*** | ***Precision*** | ***Number Needed to Read (NNR)*** |
| Centre for Reviews and Dissemination (CRD) | 1. meta analysis/  2. systematic review/  3. systematic review.pt.  4. (metaanaly$ or meta-analy$).tw.  5. metanal$  6. nursing interventions.pt.  7. (review$ or overview$).ti.  8. literature review/  9. exp literature searching/  10. cochrane$.tw.  11. synthes$.tw. adj3 (literature$ or research$ or studies or data).tw.  12. (MEDLINE or medlars or embase or scisearch or psycinfo or psychinfo or psyclit or psychlit).tw,sh.  13. pooled analy$.tw.  14. ((data adj2 pool$) and studies).tw.  15. ((hand or manual$ or database$ or computer$) adj2 search$).tw.  16. reference databases/  17. ((electronic$ or bibliographic$) adj2 (database$ or data base$)).tw.  18. (review or systematic-review or practice-guidelines).pt.  19. (review$ or overview$).ab.  20. (systematic$ or methodologic$ or quantitativ$ or research$ or literature$ or studies or trial$ or effective$).ab.  21. 18 and 20  22. 19 adj10 20  23. or/1-17,21,22  24. editorial.pt.  25. letter.pt.  26. case study.pt.  27. record review/  28. peer review/  29. (retrospective$ adj2 review$).tw.  30. (case$ adj2 review$).tw.  31. (record$ adj2 review$).tw.  32. (patient$ adj2 review$).tw.  33. (patient$ adj2 chart$).tw.  34. (peer adj2 review$).tw.  35. (chart$ adj2 review$).tw.  36. (case$ adj2 report$).tw.  37. exp case control studies/  38. exp prospective studies/  39. case studies/  40. animal studies/  41. "edit and review"/  42. (rat$ or mouse or mice or hamster$ or animal$ or dog$ or cat$ or rabbit$ or bovine or sheep).tw.  43. or/24-42  44. 43 not (43 and 23)  45. 23 not 44 | 98.4 (94.5, 99.6) | 94.0 (94.0, 94.0) | 0.8 (0.7, 0.8) | 130.4  (128.9, 136.2) |
| Wong (Best sensitivity) | 1. meta-analysis.mp.  2. review.pt.  3. systematic review.pt.  4. or/1-3 | 96.1 (91.2, 98.3) | 94.6 (94.6, 94.6) | 0.8 (0.8, 0.8) | 120.8  (118, 127.7) |
| health-evidence.ca Systematic Review search filter | 1. MEDLINE.tw.  2. exp systematic review/ or systematic review.tw  3. meta analysis/  4. intervention$.ti  5. or/1-4 | 89.9 (93.5, 94.0) | 97.6 (97.6, 97.6) | 1.8 (1.6, 1.8) | 57.2  (54.7, 61.7) |
| McKibbon (1998) | 1. meta-analysis.pt. or meta-analysis/ or meta-analysis.tw or metaanalysis.tw  2. systematic review.tw  3. (MEDLINE.tw. OR review.pt.)  4. or/1-3 | 78.3 (70.5, 84.5) | 98.9 (98.9, 98.9) | 3.2 (2.9, 3.4) | 31.7  (29.3, 35.2) |
| Wong (Best Specificity) | 1. meta-analysis.tw.  2. systematic review.pt.  3. or/1-2 | 47.3 (38.9, 55.8) | 99.4 (99.4, 99.4) | 3.5 (2.8, 4.1) | 29.1  (24.7, 35.5) |
| Wong (Small drop in sensitivity, with substantive gain in specificity) | 1. meta analysis.mp.  2. review.pt.  3. or/1-2 | 45 (36.7, 53.6) | 95.3 (95.3, 95.3) | 0.5 (0.4, 0.5) | 235.3  (193.9, 296.4) |
| Wong (Best optimization) | 1. confidence intervals.sh.  2. dt.fs.  3. review.pt.  4. or/1-3 | 50.4 (42, 58.8) | 99.4 (99.4, 99.4) | 3.8 (3.2, 4.5) | 26.3  (22.5, 31.6) |
| health-evidence.ca Public Health filter | 1. exp health promotion/  2. exp health education/  3. primary prevention.mp.  4. exp public health/  5. exp community health services/ or exp preventive health care/  6. prevention.mp.  7. exp education/  8. or/1-7  9. exp Meta Analysis/ or exp systematic Review/ or review.pt.  10. 8 and 9 | 37.2 (29.4, 45.8) | 98.2 (98.2, 98.2) | 1 (0.8, 1.2) | 107.8  (86.8, 138.6) |
